# Social capital and STIs testing among young men in Stockholm, Sweden: A cross-sectional study

**DOI:** 10.1093/eurpub/ckac130.113

**Published:** 2022-10-25

**Authors:** APF Canabarro, M Eriksson, A Nielsen, M Salazar

**Affiliations:** 1 Department of Global Public Health, Karolinska Institutet, Stockholm, Sweden; 2 Department of Social Work, Umeå University, Umeå, Sweden

## Abstract

**Background:**

Testing for sexually transmitted infections (STIs) is highly accessible in Sweden, but young men's testing rate is considerably lower than young women's. Social capital (SC) might shape people's STIs testing patterns. However, such association has not been studied among young men before. This study assessed the prevalence of different forms of SC and if they increase STIs testing among young men in Stockholm, Sweden.

**Methods:**

This population-based cross-sectional study was conducted in 2018 and included 523 men aged 20-29 years living in Stockholm. Bonding SC (having helped someone; having received help; having someone to share inner feelings with), institutionalized trust SC (in school; healthcare; media) and STIs testing behavior (never tested, tested only in the last 12 months, only more than 12 months ago, or both before and after the last 12 months) were assessed. Weighted adjusted multinomial logistic regression tested the associations between SC and STIs testing.

**Results:**

High levels of bonding SC (range: 86.5 - 95.5%), as well as trust in healthcare (76.7%) and school (64.8%) were reported. Having helped someone (aRRR 6.1, 95% CI 1.7 - 21.6), having received help (aRRR 8.1, 95% CI 2.6 - 24.7) and having someone to share feelings (aRRR 4.0, 95% CI 1.7 - 9.2) were associated with being tested for STIs more than 12 months ago. Trust in media was the only institutional trust significantly associated with STIs testing (tested in the last 12 months: aRRR 2.5, 95% CI 1.1 - 5.4; both before and after: aRRR 3.8, 95% CI 1.6 - 8.9).

**Conclusions:**

Peer-to-peer interventions using bonding SC should be used to promote STIs testing. More studies are needed to understand how trust in media increases testing for STIs. Although trust in healthcare and school were not statistically associated with testing, the high overall trust in these institutions reported in our study could be harnessed to implement sexual education programs promoting STIs testing among young men.

**Key messages:**

- Higher level of bonding social capital, as well as trusting in media, were positively associated with being tested for STIs among young men in Stockholm.
- The participants’ reported high institutional trust in healthcare and media could be harnessed to implement sexual education programs promoting STIs testing among young men.

